# 

**Published:** 1897-04-24

**Authors:** 


					THE HOSPITAL. ABSOLUTELY PURE, therefore BEST. April 24th, 1897. /
OADBURY'S ^ ww CADBURY'S
COCOA HI Jijp COCOA
' The standard of highest purity at present JpL cSz ^ NO ALKALIES USED,
attainable."?Lancet.
URIYICH HOSPUAi.
No. 552. Yol. XXII. Lfgggg] Saturday,
April 24th, 1897.
^Subscription lenns, p?JCE TWOPENCE.
!_ 8G6 p. OD. _J

				

